# Clinical Disease Severity of Respiratory Viral Co-Infection versus Single Viral Infection: A Systematic Review and Meta-Analysis

**DOI:** 10.1371/journal.pone.0099392

**Published:** 2014-06-16

**Authors:** Sandra A. Asner, Michelle E. Science, Dat Tran, Marek Smieja, Arnaud Merglen, Dominik Mertz

**Affiliations:** 1 Division of Infectious Diseases, Department of Paediatrics, The Hospital for Sick Children, University of Toronto, Toronto, Ontario, Canada; 2 Department of Clinical Epidemiology and Biostatistics, McMaster University, Hamilton, Ontario, Canada; 3 Department of Medicine, McMaster University, Hamilton, Ontario, Canada; 4 Department of Pathology and Molecular Medicine, McMaster University, Hamilton, Ontario, Canada; 5 Division of Paediatric Medicine and the Paediatric Outcomes Research Team, The Hospital for Sick Children, University of Toronto, Toronto, Canada; 6 Division of General Paediatrics, University Hospitals of Geneva and Faculty of Medicine, University of Geneva, Geneva, Switzerland; 7 Unit of Paediatric Infectious Diseases, Department of Paediatrics, University Hospital Lausanne, Lausanne, Switzerland; 8 Division of Infectious Diseases, Department of Medicine, University Hospital Lausanne, Lausanne, Switzerland; 9 Hamilton Health Sciences, Hamilton, Ontario, Canada; 10 Michael G. DeGroote Institute for Infectious Diseases Research, McMaster University, Hamilton, Ontario, Canada; Kliniken der Stadt Köln gGmbH, Germany

## Abstract

**Background:**

Results from cohort studies evaluating the severity of respiratory viral co-infections are conflicting. We conducted a systematic review and meta-analysis to assess the clinical severity of viral co-infections as compared to single viral respiratory infections.

**Methods:**

We searched electronic databases and other sources for studies published up to January 28, 2013. We included observational studies on inpatients with respiratory illnesses comparing the clinical severity of viral co-infections to single viral infections as detected by molecular assays. The primary outcome reflecting clinical disease severity was length of hospital stay (LOS). A random-effects model was used to conduct the meta-analyses.

**Results:**

Twenty-one studies involving 4,280 patients were included. The overall quality of evidence applying the GRADE approach ranged from moderate for oxygen requirements to low for all other outcomes. No significant differences in length of hospital stay (LOS) (mean difference (MD) −0.20 days, 95% CI −0.94, 0.53, p = 0.59), or mortality (RR 2.44, 95% CI 0.86, 6.91, p = 0.09) were documented in subjects with viral co-infections compared to those with a single viral infection. There was no evidence for differences in effects across age subgroups in *post hoc* analyses with the exception of the higher mortality in preschool children (RR 9.82, 95% CI 3.09, 31.20, p<0.001) with viral co-infection as compared to other age groups (*I^2^* for subgroup analysis 64%, p = 0.04).

**Conclusions:**

No differences in clinical disease severity between viral co-infections and single respiratory infections were documented. The suggested increased risk of mortality observed amongst children with viral co-infections requires further investigation.

## Introduction

Respiratory viral co-infections, defined as the detection of more than one viral pathogen in the same sample are detected in up to 30% of children with an acute respiratory tract infection (ARI) [1], [2]. While respiratory syncytial virus (RSV), influenza (INF) and human metapneumovirus (hMPV) have been mainly identified amongst children with single viral infections, other viruses including adenovirus (ADV), coronavirus and human rhinovirus (HRV) have been mainly reported amongst children with viral co-infections [2].

A better understanding of the effect of viral co-infections on disease severity is needed, considering the associated burden of respiratory viral infections. No systematic review on this topic has been published to date. A non-systematic and narrative review summarized eight cohort studies using either conventional techniques or molecular assays for detection of viruses [3]. The authors reported increased hospitalization rates among patients with viral co-infections compared to single respiratory viral infections (46.3% vs. 21.7%, p<0.01) suggesting increased severity with viral co-infections. These findings were limited, however, by including heterogeneous patient-populations (adults and children, with or without underlying comorbid conditions) and by combining studies using conventional as well as molecular methods for viral detection and by a potentially biased selection of the included studies.

We conducted a systematic review and meta-analysis to assess the association between viral status (i.e., single viral versus viral co-infection) as detected by molecular assays and the severity of clinical disease in children as well as adults hospitalized with an ARI. A systematic and comprehensive review of available literature was performed by using a transparent and systematic approach in searching, assessing and summarizing all the available evidence on this topic. We also aimed to explore whether heterogeneity in results was in part explained by different patient populations through subgroups analyses.

## Materials and Methods

This meta-analysis was performed in accordance to a protocol developed *a priori*. We reported methods and findings according to PRISMA guidelines [4].

### Eligibility criteria

We included observational studies reporting on patients of any age admitted to hospital with an acute respiratory viral illness documented by molecular assays and comparing clinical disease severity between patients with viral co-infections to those with a single viral respiratory illness, as defined below. We excluded studies which only reported data on outpatients, used multiple diagnostic techniques for viral detection, or included viral-bacterial co-infections.

### Primary and secondary outcomes

The primary outcome reflecting clinical disease severity was length of hospital stay (LOS). This outcome is commonly used as a proxy of disease severity as it is thought to reflect well the duration of illness and is highly associated with health costs [5]. We selected *a priori* four secondary outcomes, which included admission to the intensive care unit, need for mechanical ventilation, oxygen requirements and mortality.

### Literature search and data extraction

We searched MEDLINE, EMBASE, the Cochrane Central Register of Controlled Trials, CINAHL and HTA (Health Technology Assessment) for relevant studies published any year or in any language up to January 28, 2013. The search strategy was created in collaboration with a librarian (Appendix S1 in File S1). We also searched conference proceedings from 2008 to 2013 using the Web of Science and Open-SIGLE databases, and we searched Clinical trial registries (clinicaltrials.gov) and Current Controlled Trials (controlled-trials.com). We also reviewed reference lists of key articles.

Two reviewers (M.E.S and S.A.A) independently screened the titles and abstracts. Potentially relevant full text articles were then screened independently for eligibility. Agreement was calculated using Cohen's kappa coefficient [6]. Disagreements were resolved by consensus discussion or third-party adjudication (A.M). Authors were contacted for additional information when required. Reviewers independently abstracted data using a standardized form.

### Assessment of risk of bias and overall quality evidence

Two reviewers who had content and methodological expertise independently and in duplicate assessed and graded the risk of bias for included studies with a previously used, adapted version [7] of the Newcastle-Ottawa scale (NOS), which has been described elsewhere [8]. The maximum score was 8, the minimum score 0. It was decided *a priori* that a score of 7 was reflective of high methodological quality (e.g., low risk of bias), a score of 5 or 6 indicated moderate quality and a score of 4 or less indicated low quality (e.g., high risk of bias). Assessment was conducted by both reviewers independently. Publication bias was assessed by visual inspection of funnel plot.

The quality of evidence was independently assessed for each outcome by 2 reviewers according to the GRADE framework (Grading of Recommendations Assessment, Development and Evaluation) [9]. Disagreements were resolved as described above. Because randomized controlled trials addressing this research question are not feasible, observational studies were considered as the highest quality of evidence. The GRADE profiler (GRADEpro) [10] was used to present summary tables (Table 1).

**Table 1:** Summary of findings for the severity of clinical disease of children with viral co-infections versus single respiratory acute illnesses.

Outcomes	Illustrative comparative risks (95% CI)		Relative effect (95%)	N of Participants (studies)	Quality of the evidence (GRADE)
	Assumed risk (Control group)	Corresponding risk (with viral co-infections)			
Length of hospital stay			**−0.20 days** (−0.94 to 0.53)	2153 (11)	low^a^^,^ b ^,^ c ^,^ d
Oxygen requirements	319 per 1000	316 per 1000 (249 to 402 per 1000)	RR 0.99 (0.78 to 1.26)	1926 (8)	moderate^e^^,^ f ^,^ g ^,^ h
**ICU admission**	**158 per 1000**	114 per 1000 (63 to 202 per 1000)	RR 0.72 (0.40 to 1.28)	1093 (7)	low^i^ **^,^** j **^,^** k **^,^** l
Mortality	74 per 1000	181 per 1000 (64 to 511)	RR 2.44 (0.86 to 6.91)	796 (5)	low^m^^,^ n ^,^ o
Mechanical Ventilation	472 per 1000	746 per 1000 (288 to 1000)	RR 1.58 (0.61 to 4.13)	285 (3)	low^p^^,^ q ^,^ r ^,^ s

Note: N = number, CI = confidence interval, GRADE [31] = Grading of Recommendations Assessment, Development and Evaluation, RR = risk ratio, SD = standard deviation, CI = confidence interval.

GRADE Working Group grades of evidence.

**High quality:** Further research is very unlikely to change our confidence in the estimate of effect.

**Moderate quality:** Further research is likely to have an important impact on our confidence in the estimate of effect and may change the estimate.

**Low quality:** Further research is very likely to have an important impact on our confidence in the estimate of effect and is likely to change the estimate.

**Very low quality:** We are very uncertain about the estimate.

a Serious design limitations: Eight [15], [19], [21], [32], [22], [35], [23] [24] of the eleven studies had serious design limitations including the absence of notification or adjustment for important prognostic factors and the absence of evidence provided on the similarity of co-interventions between both groups.

b Serious Inconsistency: There was high statistical heterogeneity (*I^2^* = 72%, p<0.001), which was not explained on further subgroup analysis.

c Serious indirectness of the comparison: Only three [33] [19] [24] of the eleven studies focused primarily on the comparison of disease severity between single vs viral co-infections.

d No serious imprecision: The assumption of our *a priori* hypothesis of no difference was met. Cumulative sample size was appropriate. The optimal information size to detect a 1-day difference in LOS (alpha 0.05, 90% power) assuming a mean of 4 days (standard deviation 2 days) was 85 subjects per group. The number of subject exceeded this number. The 95% CI interval was narrow and it did not cross the minimally important difference of 1 day although it included the null effect.

e Serious limitations in design. All the eight studies had serious design limitations including the absence of notification or adjustment for important prognostic factors and the absence of evidence provided on the similarity of co-interventions between both groups.

f Moderate inconsistency: There was moderate statistical heterogeneity (*I^2^ = *47%, p = 0.06), which was not explained on further subgroup analysis.

g No serious indirectness: Three [21] [15] [23] of the eight studies included did not focus primarily on the comparison of disease severity between single vs viral co- infections.

h No serious imprecision: Cumulative sample size was appropriate. The 95% CI interval was narrow and included the null effect as predicted in our *a priori* hypothesis.

i Serious design limitations: All but one [34] of the seven studies of had very serious design limitations including the absence of notification or adjustment for important prognostic factors and the absence of evidence provided on the similarity of co-interventions between both groups.

j Serious inconsistency: There was high statistical heterogeneity (*I^2^ = *53%, p = 0.05), which was not explained on further subgroup analysis.

k No serious indirectness: Three [21] [23] [24] of the seven studies included did not focus primarily on the comparison of disease severity between single vs viral co-infections.

l No serious imprecision: Cumulative sample size was appropriate. The 95% CI interval was narrow and included the null effect as predicted in our *a priori* hypothesis.

m Serious design limitation: all five studies had serious design limitations including the absence of notification or adjustment for important prognostic factors and the absence of evidence provided on the similarity of co-interventions between both groups.

n Serious indirectness: Two [16] [15] of the five studies included did not focus primarily on the comparison of disease severity between single vs viral co-infections.

o There was no serious imprecision: Adequate cumulative sample size, the 95%CI included the null effect as predicted in our *a priori* hypothesis although the 95%CI were wide.

p Serious design limitations: Two of the three studies [17] [23] had serious design limitations including the absence of notification or adjustment for important prognostic factors and the absence of evidence provided on the similarity of co-interventions between both groups.

q Serious Inconsistency: There was high statistical heterogeneity (*I^2^* = 75%, p = 0.05), which was not explained on further subgroup analysis.

r Serious indirectness as two [34] [23] of the three studies did not primarily focus on the comparison between single vs viral co-infections.

s Serious imprecision: Although the *a priori* hypothesis of no effect was met, serious imprecision was attributed to the inadequate cumulative sample size and large CI.

### Statistical analysis

Statistical analysis was performed using *Review Manager* 5.1 (Cochrane Collaboration) [11]. Random-effects models were used to obtain summary estimates for all outcomes as heterogeneity across studies was expected. For continuous outcomes, we used the inverse variance method to combine results. If means and standard deviations were not reported and remained unavailable after contacting authors, the median was used to reflect the mean and the standard deviation calculated by dividing the interquartile range (IQR) by 1.35 [12]. For dichotomous outcomes, the risk ratio with its 95% confidence interval (CI) was reported. Heterogeneity was evaluated using the *I* ^2^ statistic.

### Subgroup Analysis

When substantial heterogeneity was found (*I* ^2^≥40%), *a priori* defined subgroup analyses were performed which included age (<18 years versus ≥18 years), the nature of the underlying respiratory illness (ARIs, bronchiolitis, radiologically confirmed community acquired pneumonia (CAP), respectively), the presence of underlying co-morbidities, the impact of overall viral infections versus specific viral pathogens, and high versus low risk of bias in the included studies as defined above. Interaction tests for subgroup differences were conducted using the Chi-square and *I^2^* statistic. Subgroup credibility was examined using the criteria described by Sun and colleagues [13]. We made a post-hoc stratified analysis of “children” by age subgroups based on data availability in the abstracted studies (infants 0–23 months of age, preschool children 0–59 months of age and children 0–17 years old).

### Sensitivity analysis

We also evaluated the robustness of the pooled estimate related to the potential differences in disease severity described for infections with more pathogenic viruses such as INF, RSV or hMPV. Therefore we performed a *post hoc* sensitivity analysis in which only studies involving these viral infections were included [1],[2].

## Results

**Figure 1:**
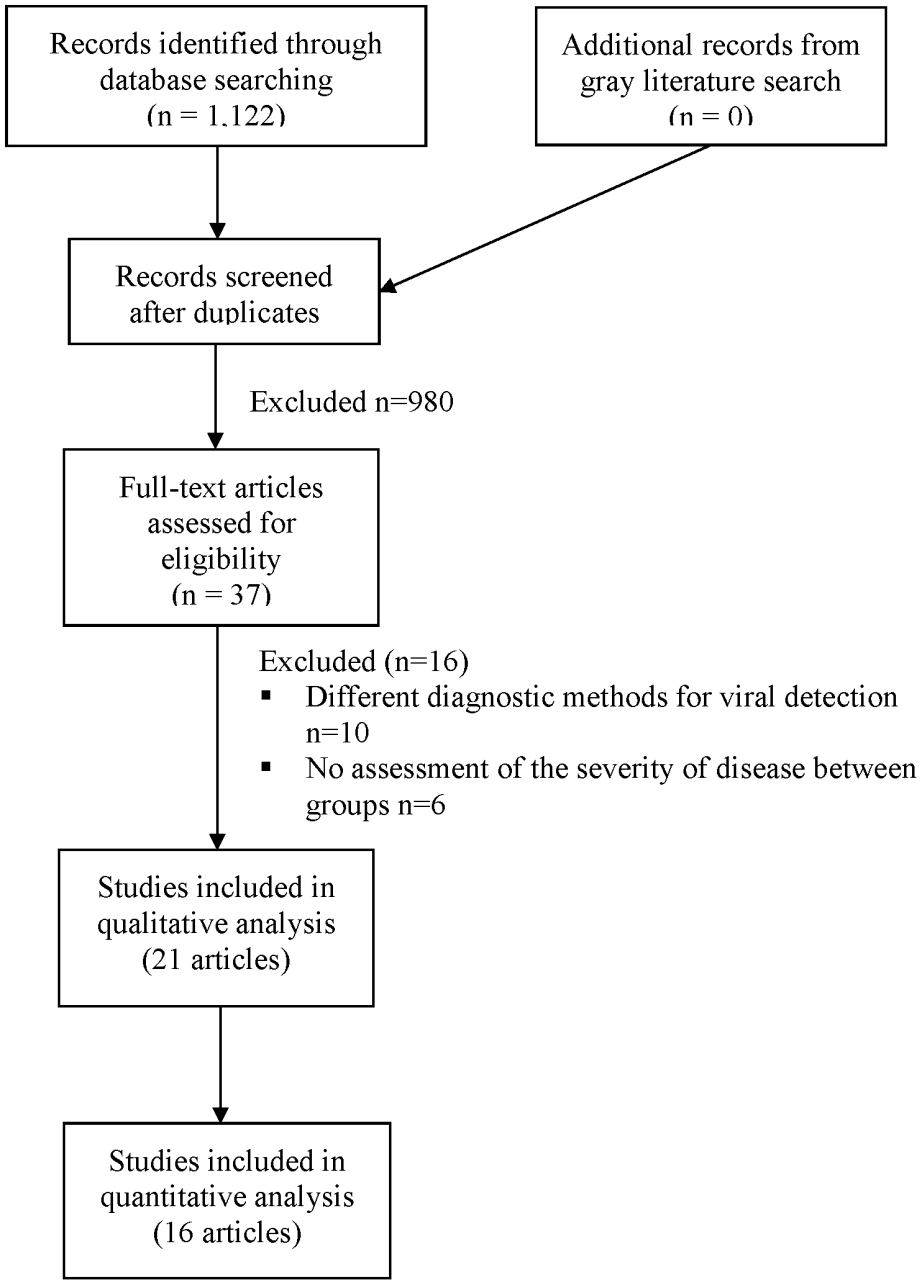
Selection of studies for the qualitative and quantitative analyses. n = number.

We screened the titles and abstracts of 1,017 unique records identified through our literature search. Of these, 37 were assessed for eligibility in full text, 21 studies met inclusion criteria (all cohort studies) of which 16 (76.1%) provided data for quantitative analysis (Figure 1). The Kappa for agreement between the reviewers was 0.96 for both screening of titles/abstracts and of full text articles. Of the 13 authors contacted, eleven [14] [15] [16] [17] [18] [19] [20] [21] [22] [23] [24] provided additional information on outcomes of interest. No additional sources were identified in the grey literature search.

### Study Characteristics

The 21 studies included reported on 4,280 patients ranging from 1 to 65 years of age (Table 2). Seventeen (80.9%) studies were exclusively conducted in children <18 years, two studies were conducted in adults and the remaining two in adults and children. Any ARI was assessed in nine studies; bronchiolitis, bronchitis and CAP in three studies each; and acute respiratory wheezing and influenza-like illness (ILI) in one study each. One study did not specify the type of respiratory illness.

**Table 2:** Characteristics of observational studies included in the qualitative analysis.

Author	Year	Country	N	Population	Co-morbidities	Samples analyzed	Molecular assays	Reported outcomes			
								LOS	Death	ICU	Oxygen req.
Aramburo [35]	2011	US	80	Children <5yo	IC, C-R	NPs	RT-PCR	yes	yes	no	no
Camargo [36]	2012	Brazil	41	Adults & Children	IC, C-R, Other	NPs	RT-PCR	no	yes	yes	no
Calvo [33]	2008	Spain	172	Children <2yo	C-R, Other	NPAs	RT-PCR	yes	no	no	no
Canducci [1]	2008	Italy	150	Children <2yo	Undefined conditions	NPAs	RT-PCR	yes	no	no	yes
Choi [16]	2010	S. Korea	72	Adults	IC, C-R, Other	NPs and BAL	RT-PCR	no	yes	no	no
Cilla [37]	2008	Spain	178	Children <3yo	IC, C-R, Other	NPAs	RT-PCR kit, Qiagen	yes	no	no	yes
De Paullis [34]	2011	Brazil	176	Children <2yo	C-R, Other	NPAs	RT-PCR	yes	yes	yes	no
Do [21]	2011	Vietnam	222	Children <15yo	None	NPAs, TS, NS	RT-PCR kit, Seeplex	yes	no	yes	yes
Esper [17]	2011	US	226	Adults & Children	None	NPAs	RT-PCR kit, Invitrogen	no	no	no	yes
Franz [18]	2010	Germany	375	Children <16yo	C-R, Other	NPAs	RT-PCR	yes	no	no	yes
Garcia Garcia [32]	2006	Spain	69	Children <2yo	C-R, Other	NPAs	RT-PCR kit, Qiagen OneStep	yes	no	no	no
Garcia Garcia [38]	2010	Spain	539	Children <14yo	Other	NPAs	RT-PCR kit, Qiagen OneStep	yes	no	no	yes
Gerna [22]	2008	Italy	47	Children <2yo	None	NPAs	RT-PCR	yes	no	no	no
Guerrier [15]	2013	Cambodia	551	Children <5yo	C-R	NPAs	RT-PCR kit, Biomérieux	yes	yes	no	yes
Marcos [39]	2009	Spain	181	Adults	IC, C-R, Other	NPs and OPs	xTAG RVP FAST, Luminex	yes	no	no	no
Marguet [40]	2009	France	126	Children <2yo	Other	NPAs	RT-PCR	yes	no	no	no
Martin [19]	2012	US	566	Children	IC, C-R, Other	Nasal wash	RT-PCR	yes	no	yes	yes
Nascimento [14]	2010	Brazil	77	Children <2yo	C-R, Other	NPAs	RT-PCR kit, Qiagen	no	no	yes	no
Nascimento [2]	2011	Brazil	11	Children <5yo	none	NPAs	RT-PCR	yes	no	no	no
Prill [23]	2012	US	113	Children <5yo	C-R, Other	Nasal & TS	RT-PCR	yes	no	yes	yes
Rhedin [24]	2012	Sweden	308	Children <17yo	IC, C-R	NPAs, NPs, BAL	RT-PCR kit, Qiagen	yes	yes	yes	no

Legend: N = number, LOS = Length of stay, ICU = admission to the intensive care unit, ILI: influenza-like illness, CAP: community acquired pneumonia; IC: immunocompromised; C-R: cardio-respiratory ARI: acute respiratory infections; NPAs: Nasopharyngeal aspirates; NPs: nasopharyngeal swabs; Ops: oropharyngeal swabs; TS: throat swabs: RT-PCR: real-time PCR. Studies, which included commercial assays (RT-PCR) were labeled as RT-PCR kit, with the mention of the manufacturer.

### Risk of Bias and Overall Quality of Evidence

Applying the adapted NOS, the studies achieved a median of 4 out of 8 points (IQR 3–4) (Table 3). The overall quality of evidence applying the GRADE approach ranged from moderate for oxygen requirements to low for all other outcomes (Table 1). One of the concerns involved serious indirectness for the LOS and the need for mechanical ventilation. For these outcomes, the majority of studies did not primarily focus on the comparison of LOS or the need for mechanical ventilation between single vs viral co-infections. A funnel plot did not suggest any publication bias for the primary outcome (Appendix S2 in File S1). Also, publication bias was judged to be minimal because of our extensive literature search, and the presence of both positive and negative studies.

**Table 3:** Risk of bias review of included studies using an adapted version of the Newcastle-Ottawa scale.

Study	Groups from the same population	Exposure assessment	Matching	Prognostic factors assessment	Outcome assessment	Adequacy of follow-up	Similar co-interventions	Total points
Aramburo [35]	1	1	0	1	1	1	0	5
Calvo [33]	1	1	0	1	1	1	0	5
Camargo [36]	1	1	0	1	1	0	0	4
Canducci [1]	1	1	0	0	1	1	0	4
Choi [16]	1	1	0	1	1	0	0	4
Cilla [37]	1	1	0	0	1	0	0	3
De Paullis [34]	1	1	0	1	1	1	1	6
Do [21]	1	1	0	0	1	0	0	3
Esper [17]	1	0	0	0	1	1	0	3
Franz [18]	1	1	0	0	1	1	0	4
Garcia Garcia [38]	1	1	0	0	1	1	0	4
Garcia Gracia [32]	1	1	0	0	1	1	0	4
Gerna [22]	1		0	0	1	1	0	3
Guerrier [15]	1	1	0	0	1	1	0	4
Marcos [39]	1	1	0	1	1	1	0	5
Marguet [40]	1	0	0	1	1	1	0	4
Martin [19]	1	1	0	0	1	0	0	3
Nascimento et al [14]	1	1	0	0	1	1	0	4
Nascimento et al [2]	1	1	0	0	0	1	0	3
Prill et al [23]	0	0	0	0	0	0	0	0
Rhedin et al [24]	0	0	0	0	0	0	0	0

Legend:

For each column: 1: achieved, 0: not achieved. The maximum score within the total points category was 8, the minimum score 0. A score of 7 represented high quality, a score 5–6: moderate quality and a score ≤4: low quality.

Relevant subgroup analyses were conducted for the length of hospital stay and mortality as stated under these outcomes.

### Primary outcome: length of hospital stay (LOS)

**Figure 2:**
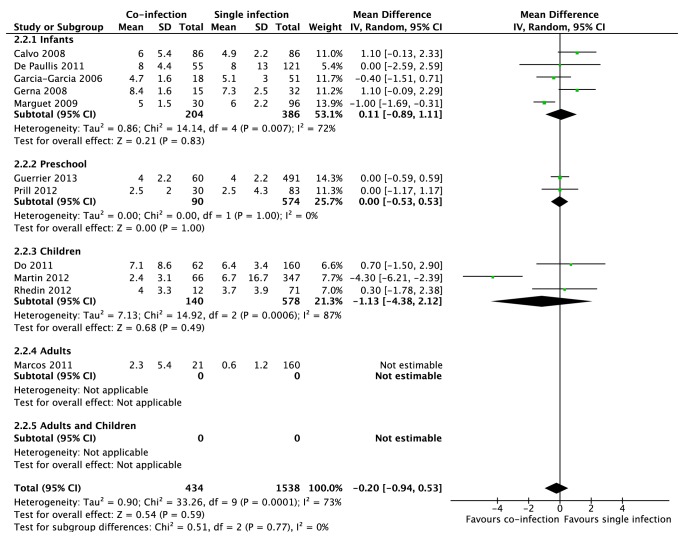
Meta-analysis of the length of stay, by age groups (infants, preschool children, children, adults and adults and children) between patients with viral coinfections and single viral infections. A value less than zero indicates a shorter length of hospital stay (number of days). in patients co-infected with more than virus (favors co-infection). CI = confidence interval, df = degrees of freedom. Age groups: Infants 0–23 months of age, Preschool children 0–59 months of age and Children 0–17 years old.

Eleven studies involving 2,531 patients reported the LOS and were pooled in meta-analysis. There was no significant difference in length of admission between groups (mean difference (MD) −0.20 days, 95% CI −0.94, 0.53, p = 0.59). (Figure 2) Because of significant heterogeneity (*I^2^ = *73%, p<0.001), inconsistency and indirectness of the comparison documented for this outcome, the quality of evidence was downgraded to low.

Some of the heterogeneity could be explained by the type of respiratory illnesses: subgroup analyses showed a significantly shorter LOS stay among children with bronchiolitis and viral co-infection (MD −0.83 days; 95%CI −1.42, −0.24; p = 0.006) while no differences between viral groups were observed amongst subjects with ARI, although the interaction test was not significant (p = 0.10) (Appendix S3 in File S1). In post-hoc age subgroup analyses, the effect was similar across all age subgroups (*I^2^* for subgroup analysis 0%, p = 0.77) (Figure 2).

Sensitivity analysis including only studies on viruses with a higher pathogenic potential (RSV A, RSV B, INF A, INF B, PIV and hMPV) revealed similar results (MD 0.24 days, −0.64, 1.12, p = 0.59) (Appendix S4 in File S1).

### Secondary outcomes

**Figure 3:**
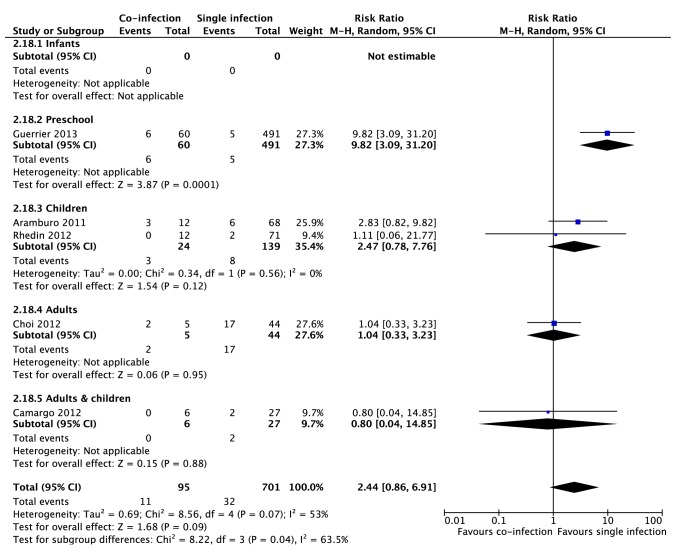
Meta-analysis of mortality between patients with viral co-infections and single viral infections by age groups (infants, preschool children, children, adults and adults and children). A value higher than one indicates a higher mortality in patients co-infected with more than virus (favors single infection). CI = confidence interval, df = degrees of freedom. Age groups: Infants 0–23 months of age, Preschool children 0–59 months of age and Children 0–17 years old.

Five studies provided data for meta-analysis on mortality including a total of 1,794 patients. There was a trend of higher mortality rates among patients with viral co-infections (RR 2.44, 95% CI 0.86, 6.91, p = 0.09) with a high degree of heterogeneity (*I^2^* = 53%). Although there was a significant subgroup effect (*I^2^* for subgroup analysis = 63%, p = 0.04) with higher mortality in preschool children with viral co-infections (RR 9.82, 95% CI 3.09–31.2, p<0.001), this was based on only one study, which was found to be at a high risk of bias (Figure 3).

Seven studies involving 893 patients reported the proportion of patients admitted to the ICU. No difference were observed (relative risk (RR) 0.72, 95% CI 0.40, 1.28, p = 0.26) with high heterogeneity (*I^2^* = 53%). Also, there were no differences between age subgroups (*I^2^* for subgroup analysis = 0%, p = 0.50) (Appendix S5 in File S1).

Only three studies including 515 patients provided detailed data on the need for mechanical ventilation. Again, there was no significant difference between groups (RR 1.58, 95% CI 0.61, 4.13, p = 0.35) but heterogeneity was high (*I^2^* = 75%). Although there was a statistically significant difference in age group effects (*I^2^* for subgroup analysis = 74%, p = 0.05), this was based on only two studies, one in infants with more benign outcomes (RR 1.01, 95% CI 0.57–1.78, p = 0.98) compared to one study in a mixed population (RR 2.69, 95% CI 1.22–5.94, p = 0.01) (Appendix S6 in File S1).

Eight studies involving 2,294 patients provided data for the meta-analysis on oxygen requirement as an outcome. No difference was found between both groups with high heterogeneity (RR 0.99, 95% CI 0.78, 1.26, p = 0.94, *I^2^* = 47%). In post-hoc age subgroup analysis, the effect was similar across all age subgroups (for subgroup analysis = 48.5%, p = 0.12) (Appendix S7 in File S1).

## Discussion

While we found no overall differences in clinical severity between patients admitted with single viral respiratory illnesses and those with viral co-infections, preschool children with co-infections might be at increased risk of death. Although our findings were consistent across outcomes, they were tempered by significant heterogeneity.

We found no significant difference between groups with respect to the LOS, admission to the ICU, the need for mechanical ventilation and oxygen requirements as suggested in the majority of recently published studies [25]. However, while viral co-infection had little impact on mortality in one of these studies including adult patients admitted to an intensive care unit (ICU) in Australia [25] with pH1N1 infection, co-infection with seasonal influenza A and influenza B viruses was associated with a significant increase in risk of ICU admission and death in another study [26]. These divergent findings may be explained by differences in age groups of the population studied, the range of illness severity and the proportion of patients with comorbid conditions, seasonal differences regarding circulating respiratory viruses and performance of different molecular panels used [27].

We found a significantly higher risk of death in children with viral co-infections when compared to single infections. The single study in adults [16], however, did not find a difference, while the effect in children was primarily driven by one study [15]. Of note, this statement was not supported by findings across other outcomes, and, in addition, there was moderate statistical heterogeneity and the interaction test was not significant thus limiting the conclusions from this subgroup analysis.

There was no evidence for differences in effects across age subgroups in *post hoc* analyses with the exception of the higher mortality in preschool children as compared to other age groups. One may have expected differences in how co-infections affect patient outcomes based on a recent experimental study assessing the course of infection of young versus old mice which were infected with RSV and/or HMPV. They observed that the elevation in TNF-α and NF-κB was influenced by the age of the mice, the type of virus (i.e RSV and hMPV) and single versus co-infection (28). While these finding suggest that age may influence the severity of co- versus single virus infection, the published clinical evidence summarized in this systematic review could not corroborate this assumption. Future studies conducted among adults and children, with defined age subgroups, which will incorporate measures of TNF-α and NF-κB levels may better support findings observed in experimental studies.

While the pathogenicity of certain viruses such as RSV and INF is well established, uncertainty remains about the virulence of HBoV, coronaviruses and HRV. Their common detection in co-infections with other viruses and from asymptomatic subjects may result from the increased sensitivity of molecular assays [2]. This is supported by the significantly lower viral loads of these viruses when detected in co-infection with INF or RSV [28]. The increased severity of viral co-infections observed in previous studies using conventional techniques [3] possibly resulted from limited viral detection, which was restricted to potentially more pathogenic viruses such as RSV, INF or hMPV. However, a *post hoc* sensitivity analysis including viral co-infections with these viruses only supported the robustness of the above findings.

The mechanisms of disease virulence in co-infections are not clearly understood. They may result from direct interactions of viral genes or indirect interactions resulting from alterations in the host-environment or immunological interactions [29]. Thus, different pathogenic mechanisms may be triggered by different viruses, which may potentiate or inhibit each other's effects. As such, certain pairing of viruses may be more clinically relevant than others. Yet, viral-viral interaction is poorly understood.

### Strengths and limitations

The strengths of this systematic review include a systematic, protocol-driven and comprehensive review with extensive literature search, minimal evidence for publication bias and successful attempts to contact authors. In addition, rigorous assessment of eligibility ensured high reliability of the results. All subgroups analyses, with the exception of age subgroups analyses, were defined *a priori* and *post hoc* sensitivity analyses confirmed robustness of the results. Finally a rigorous use of the GRADE approach ensured a transparent and comprehensive approach to evaluate overall quality of the studies. Limitations predominantly relate to the high risk of bias of the included studies. Of note, no studies reported risk estimates adjusted for important prognostic factors such as underlying co-morbidities or bacterial co-infections. Although the influence of comorbid conditions was partially addressed in our subgroup analysis, an insufficient number of studies precluded some subgroup analyses we planned *a priori*. Respiratory viral infections are known to predispose to secondary bacterial pulmonary infections, and thus can result in substantial confounding [30]. Finally, age subgroup analyses were conducted *post hoc*. Based on the data available we were able to provide subgroups as defined above, but the data did not allow us to specify other, better discriminating age groups.

## Conclusions

In conclusion, we found no convincing evidence that patients admitted with viral co-infections are at higher risk for increased disease severity than patients presenting with single respiratory viral infections –with the potential exception of mortality in preschool children of <5 years of age. Large, rigorously conducted studies including multivariable analyses adjusting for important confounders are lacking, and thus, new studies would be very likely to significantly change our overall assessment. Prospective longitudinal studies, which will focus on objective outcomes and include serial respiratory sampling for viruses and bacteria may lead to a better understanding of the clinical significance of polymicrobial acute respiratory infections.

## Supporting Information

File S1 **This file includes the following: Appendix S1:** exp: respiratory, adj = adjunctive. **Appendix S2:** MD = mean difference, SE (MD) = standard error (mean difference). **Appendix S3:** A value less than zero indicates a shorter length of hospital stay (LOS) (number of days) in patients co-infected with more than one virus (favors co-infections). CI = confidence interval, df = degrees of freedom. **Appendix S4:** A value less than zero indicates a shorter length of hospital stay (LOS) (number of days) in patients co-infected with more than one virus (favors co-infections). CI = confidence interval, df = degrees of freedom. **Appendix S5:** A value higher than one indicates a higher admission rate in the ICU in patients co-infected with more than one virus (favors single). CI = confidence interval, df = degrees of freedom. Age groups: Infants 0–23 months of age, Preschool children 0–59 months of age and Children 0–17 years old. **Appendix S6:** A value higher than one indicates a higher need for mechanical ventilation in patients co-infected with more than one virus (favors single). CI = confidence interval, df = degrees of freedom. Age groups: Infants 0–23 months of age, Preschool children 0–59 months of age and Children 0–17 years old. **Appendix S7:** A value higher than one indicates higher oxygen requirements in patients co-infected with more than one virus (favors single). CI = confidence interval, df = degrees of freedom. Age groups: Infants 0–23 months of age, Preschool children 0–59 months of age and Children 0–17 years old.
(DOCX)

Checklist S1 **PRISMA Checklist.**
(DOCX)
