# Supplementary material for: Clinical Disease Severity of Respiratory Viral Co-Infection versus Single Viral Infection: A Systematic Review and Meta-Analysis
Source: PLoS One. 2014 Jun 16;9(6):e99392. doi: 10.1371/journal.pone.0099392 (PMC4059637; doi:10.1371/journal.pone.0099392)
Supplement: File S1 — This file includes the following: Appendix S1: exp: respiratory, adj = adjunctive. Appendix S2: MD = mean difference, SE (MD) = standard error (mean difference). Appendix S3: A value less than zero indicates a shorter length of hospital stay (LOS) (number of days) in patients co-infected with more than one virus (favors co-infections). CI = confidence interval, df = degrees of freedom. Appendix S4: A value less than zero indicates a shorter length of hospital stay (LOS) (number of days) in patients co-infected with more than one virus (favors co-infections). CI = confidence interval, df = degrees of freedom. Appendix S5: A value higher than one indicates a higher admission rate in the ICU in patients co-infected with more than one virus (favors single). CI = confidence interval, df = degrees of freedom. Age groups: Infants 0–23 months of age, Preschool children 0–59 months of age and Children 0–17 years old. Appendix S6: A value higher than one indicates a higher need for mechanical ventilation in patients co-infected with more than one virus (favors single). CI = confidence interval, df = degrees of freedom. Age groups: Infants 0–23 months of age, Preschool children 0–59 months of age and Children 0–17 years old. Appendix S7: A value higher than one indicates higher oxygen requirements in patients co-infected with more than one virus (favors single). CI = confidence interval, df = degrees of freedom. Age groups: Infants 0–23 months of age, Preschool children 0–59 months of age and Children 0–17 years old. (DOCX) [file pone.0099392.s001.docx]

**Appendix S1. Search strategy for studies as of January 28, 2013**

| # | **OvidSP MEDLINE (1946 to January 28, 2013)** |  |
| --- | --- | --- |
| Set | **History** | **Results** |
| 1 | respiratory tract infections/ or bronchitis/ or bronchiolitis, viral/ or common cold/ or influenza, human/ or laryngitis/ or nasopharyngitis/ or pneumonia, viral/ or rhinitis/ or exp sinusitis/ or exp supraglottitis/ or tracheitis/ or Viruses/ or exp influenzavirus a/ or exp influenzavirus b/ or influenzavirus c/ or virus diseases/ | 159948 |
| 2 | exp dna virus infections/ or exp rna virus infections/ | 598335 |
| 3 | coinfection/ or ("co infection" or coinfection* or "co-infection*" or ((mixed or secondary or polymicrobial) adj2 infection*)).ti,ab. | 21856 |
| 4 | 1 and 2 and 3 | 631863 |
| 5 | cohort studies/ or longitudinal studies/ or follow-up studies/ or prospective studies/ (889323)  12 case-control studies/ or retrospective studies/ or cross-sectional studies/ | 1453148 |
| 6 | 4 and 5 | 136 |
| 7 | microbiological techniques/ or viral load/ or (molecular adj2 assay*).ti,ab. | 25327 |
| 8 | 1 and 3 and 7 | 44 |
| 9 | 6 or 8 | 172 |

Search strategies for other databases are available upon request.

**Appendix S2. Funnel plot for the primary outcome**

**
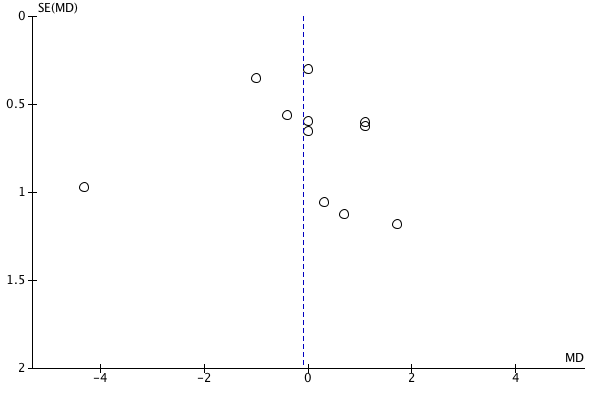
**


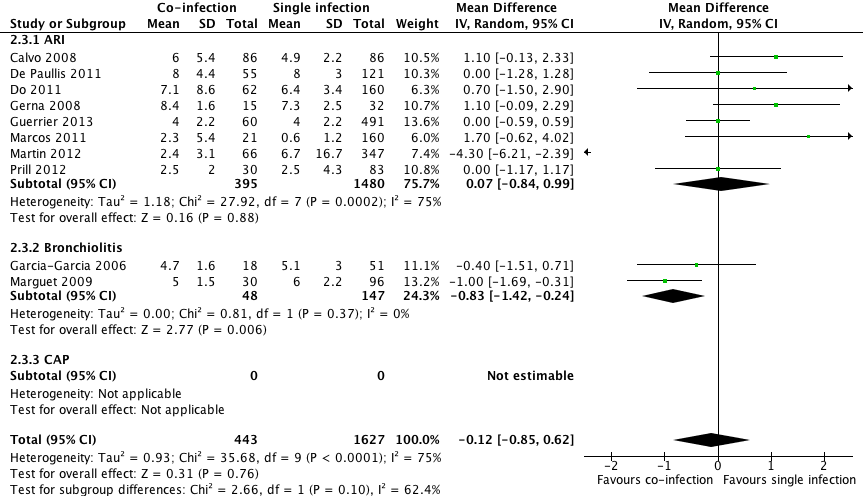
**Appendix S3. Meta-analysis of the LOS*, by type of respiratory illness, between patients with viral co-infections and single viral infections.**

Legend: A value less than zero indicates a shorter LOS in patients co-infected with more than one virus (favors co-infections). CI=confidence interval, df=degrees of freedom

***LOS: Length of hospital stay (number of days)**


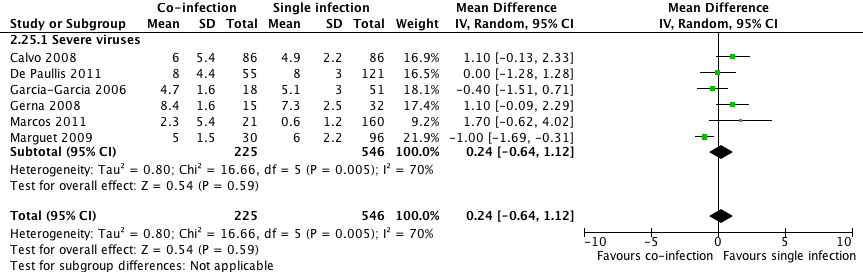
**Appendix S4. Meta-analysis of the LOS*, by more severe viral pathogens (RSV, FLU, PIV or hMPV ) between patients with viral co-infections vs single viral infections.**

Legend: A value less than zero indicates a shorter LOS in patients co-infected with more than one virus (favors co-infections). CI=confidence interval, df=degrees of freedom

***LOS: Length of hospital stay (number of days)**

**Appendix S5. Meta-analysis of admission in the intensive care unit (ICU), by age groups (infants, preschool, children, adults, adults and children) between patients with viral co-infections vs single viral infections.**


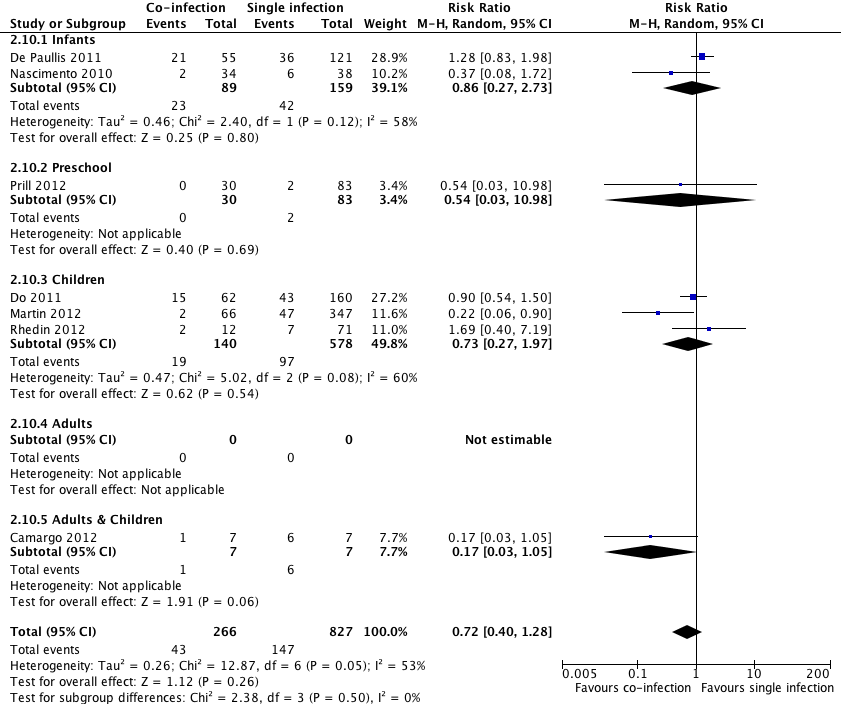


Legend: A value higher than one indicates a higher admission rate in the ICU in patients co-infected with more than one virus (favors single). CI=confidence interval, df=degrees of freedom. Age groups: Infants 0-23 months of age, Preschool children 0-59 months of age and Children 0-17 years old


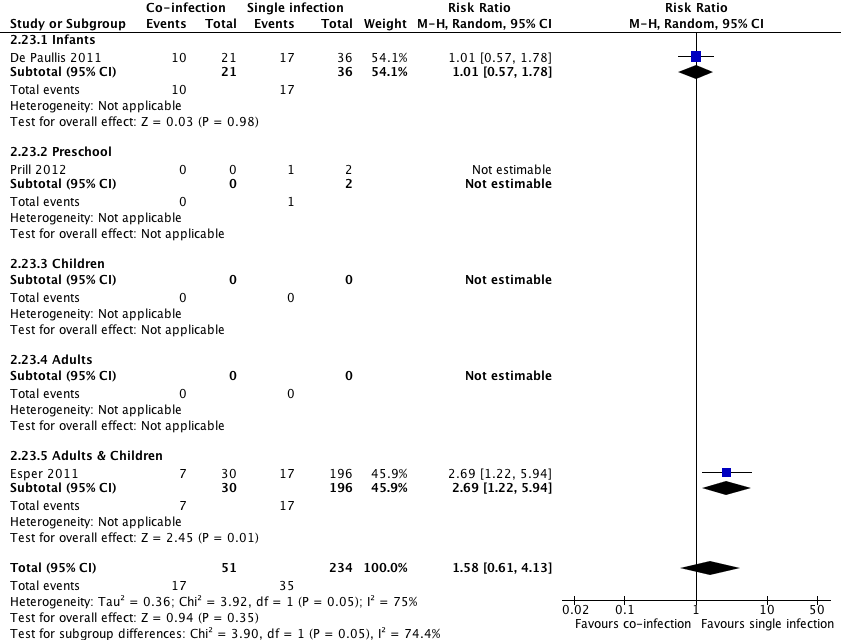
**Appendix S6. Meta-analysis of the need for mechanical ventilation among patients admitted in the ICU, by age groups (infants, preschool, children, adults, adults and children), between patients with viral co-infections vs single viral infections.**

Legend: A value higher than one indicates a higher need for mechanical ventilation in patients co-infected with more than one virus (favors single). CI=confidence interval, df=degrees of freedom. Age groups: Infants 0-23 months of age, Preschool children 0-59 months of age and Children 0-17 years old


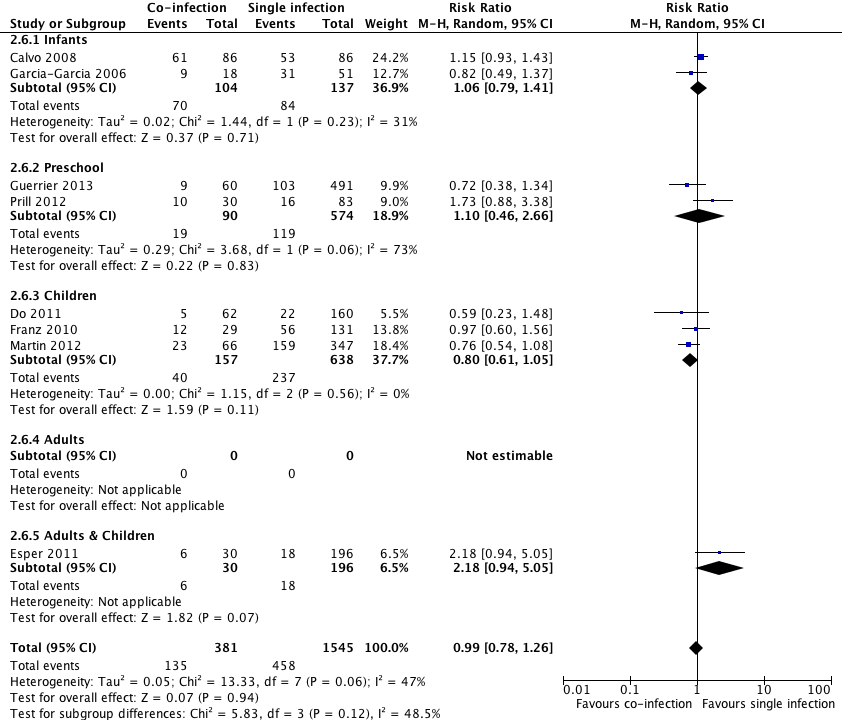
**Appendix S7. Meta-analysis of oxygen requirements, by age groups (infants, preschool, children, adults, adults and children), between patients with viral co-infections vs single viral infections.**

Legend: A value higher than one indicates higher oxygen requirements in patients co-infected with more than one virus (favors single). CI=confidence interval, df=degrees of freedom. Age groups: Infants 0-23 months of age, Preschool children 0-59 months of age and Children 0-17 years old
